# Ursodeoxycholic acid prevents ventricular conduction slowing and arrhythmia by restoring T-type calcium current in fetuses during cholestasis

**DOI:** 10.1371/journal.pone.0183167

**Published:** 2017-09-21

**Authors:** Oladipupo Adeyemi, Anita Alvarez-Laviada, Francisca Schultz, Effendi Ibrahim, Michael Trauner, Catherine Williamson, Alexey V. Glukhov, Julia Gorelik

**Affiliations:** 1 Department of Cardiovascular Sciences, National Heart and Lung Institute, Imperial College London, London, United Kingdom; 2 Institute of Reproductive and Developmental Biology, Imperial College London, London, United Kingdom; 3 Faculty of Medicine, MARA Technology University, Sungai Buloh, Malaysia; 4 Department of Medicine III, Medical University of Vienna, Vienna, Austria; 5 Women's Health Academic Centre, King's College London, London, United Kingdom; 6 Department of Medicine, University of Wisconsin-Madison School of Medicine and Public Health, Madison, Wisconsin, United States of America; Universidad de Navarra, SPAIN

## Abstract

**Background:**

Increased maternal serum bile acid concentrations in intrahepatic cholestasis of pregnancy (ICP) are associated with fetal cardiac arrhythmias. Ursodeoxycholic acid (UDCA) has been shown to demonstrate anti-arrhythmic properties via preventing ICP-associated cardiac conduction slowing and development of reentrant arrhythmias, although the cellular mechanism is still being elucidated.

**Methods:**

High-resolution fluorescent optical mapping of electrical activity and electrocardiogram measurements were used to characterize effects of UDCA on one-day-old neonatal and adult female Langendorff-perfused rat hearts. ICP was modelled by perfusion of taurocholic acid (TC, 400μM). Whole-cell calcium currents were recorded from neonatal rat and human fetal cardiomyocytes.

**Results:**

TC significantly prolonged the PR interval by 11.0±3.5% (*P*<0.05) and slowed ventricular conduction velocity (CV) by 38.9±5.1% (*P*<0.05) exclusively in neonatal and not in maternal hearts. A similar CV decline was observed with the selective T-type calcium current (*I*_Ca,T_) blocker mibefradil 1μM (23.0±6.2%, *P*<0.05), but not with the L-type calcium current (*I*_Ca,L_) blocker nifedipine 1μM (6.9±6.6%, NS). The sodium channel blocker lidocaine (30μM) reduced CV by 60.4±4.5% (P<0.05). UDCA co-treatment was protective against CV slowing induced by TC and mibefradil, but not against lidocaine. UDCA prevented the TC-induced reduction in the *I*_Ca,T_ density in both isolated human fetal (−10.2±1.5 versus −5.5±0.9 pA/pF, P<0.05) and neonatal rat ventricular myocytes (−22.3±1.1 versus −9.6±0.8 pA/pF, P<0.0001), whereas UDCA had limited efficacy on the *I*_Ca,L_.

**Conclusion:**

Our findings demonstrate that *I*_Ca,T_ plays a significant role in ICP-associated fetal cardiac conduction slowing and arrhythmogenesis, and is an important component of the fetus-specific anti-arrhythmic activity of UDCA.

## Introduction

Intrahepatic cholestasis of pregnancy (ICP) is a gestational liver disease that typically occurs in the third trimester and can lead to sudden fetal arrhythmias and intrauterine death.[1–3] Despite the incidence of cardiac arrhythmia in the fetus, maternal cardiac abnormalities are not reported in ICP.[2] Because of the obvious complexities associated with studying fetal hearts *in utero*, the mechanisms underlying ICP associated fetal arrhythmias remain poorly understood.

The maternal bile profile is altered in ICP and measurement of maternal serum bile acid concentrations is important for diagnosis and monitoring of ICP.[2;3] Cholic and chenodeoxycholic bile acids are the principal bile acid species that are elevated in ICP.[4] Of the management approaches for ICP, treatment with ursodeoxycholic acid (UDCA) is effective in restoring the maternal bile acid profile and might be beneficial in reducing the reported incidents of adverse fetal events.[2;3;5] Despite its proven efficacy, safety and wide-use, the mechanism by which UDCA is able to protect against fetal arrhythmias is still not fully understood.

Elevated plasma concentrations of taurocholic acid (TC) occur in ICP.[6] It was shown that TC causes a decline in L-type calcium current[7;8] and enhances the Na^+^-Ca^2+^ exchanger tail current density in cardiomyocytes[9] resulting in altered calcium handling and a reduced rate of contraction.[10;11] We have previously shown in an *in vitro* rat model of ICP that the pro-arrhythmic changes induced by TC are associated with a significant conduction velocity slowing, development of early afterdepolarization and reentrant arrhythmias.[12;13] These arrhythmic events were prevented by UDCA and were hypothesized to be associated with a depolarizing influence of myofibroblasts on co-cultured neonatal rat cardiomyocytes.[10;14;15] However, these findings are critically limited to an *in vitro* model of the neonatal heart, which suggests functional coupling between myofibroblasts and cardiomyocytes via heterocellular gap junctions.[10;15] Here, building on previous *in vitro* studies, we investigated the mechanisms underlying the anti-arrhythmic effect of UDCA using Langendorff-perfused neonatal and maternal hearts. To model ICP, hearts were treated with TC. We confirmed our previous *in vitro* studies that TC significantly slowed ventricular conduction velocity exclusively in neonatal, but not in maternal hearts. Importantly, we linked the conduction slowing observed in fetuses to specific downregulation of the T-type Ca^2+^ current (*I*_Ca,T_) by TC. We showed that UDCA co-treatment was protective against conduction slowing induced by TC by restoring *I*_Ca,T_. Thus, fetus-specific expression of T-type Ca^2+^ channels in ventricular myocytes could underlie their sensitivity to high bile acid concentrations and ICP-associated fetal arrhythmia. T-type Ca^2+^ channels should be considered as an important target for fetus-targeted treatment in ICP.

## Materials and methods

### Ethics statement

Human fetal hearts were obtained from surgical terminations of pregnancy at 12–17 weeks of gestation, which were terminated due to scan or genetic abnormalities, after written informed consent of the mother using Biobank ethical approval (ICHTB licence 12275, REC approval 12/WA/0196).

### Optical mapping and ECG

High-resolution optical mapping of electrical activity was performed on one-day-old neonatal rat (~7 grams, Sprague-Dawley) and adult female rat (200–400 g) hearts as previously described.[16] Hearts were randomly allocated to four treatment groups (for specific sample sizes for each group see Fig legends below). Hearts were allowed to stabilise for an initial 15-min period before being subjected for a further 15 min to either vehicle or drug treatment. Hearts were treated with increasing concentrations of TC (40, 100 and 400 μM) or vehicle. Following on from this, the effect of TC 400 μM with UDCA 1 μM or UDCA 1 μM alone were also evaluated. Comparison studies in the maternal hearts with TC were also performed at similar concentrations.

### Patch-clamp electrophysiology

For whole-cell patch-clamp studies, cardiomyocytes were isolated from one-day-old neonatal rat and human fetal hearts using enzymatic digestion as previously described.[10] The effect of vehicle, TC (100 μM) or TC (100 μM) plus UDCA (100 nM) on L- and T-type calcium currents (*I*_Ca,L_ and *I*_Ca,T_) characteristics were studied using the whole-cell patch-clamp technique.

Additional materials and methods are provided in the online supplementary information.

## Results

### UDCA protects against supraventricular conduction slowing induced by fetus-specific effect of TC

**Fig 1:**
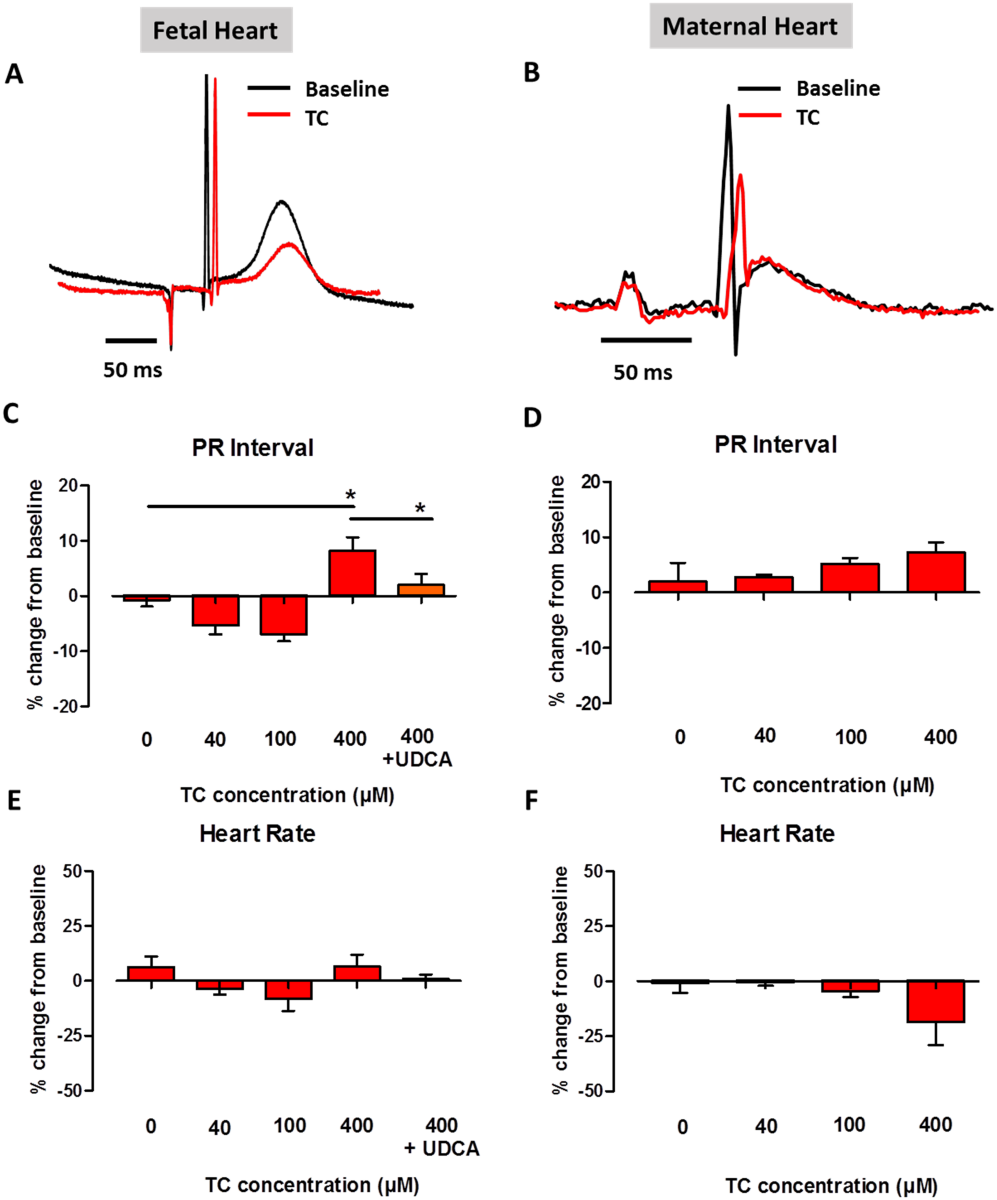
Effect of TC on PR interval and heart rate in fetal and maternal hearts. A and B: Representative ECG trace overlays following baseline and TC 400 μM treatment in fetal (left column; *n* = 3–7 per group) and maternal (right column; *n* = 3 per group) models. C and D: Plots of mean percentage change from baseline values in PR interval following treatment with TC 0, 40, 100, 400 μM and TC 400 μM with UDCA 1 μM treatment. E and F: Plots of mean percentage change from baseline values in heart rate following treatment with TC 0, 40, 100, 400 μM and TC 400 μM with UDCA 1 μM treatment. *P<0.05, One-way analysis of variance (ANOVA) and Bonferroni’s post hoc test to determine statistical significance.

On the basis of previous reports of altered heart rate and slowing of atrial impulse propagation in fetuses of ICP patients,[17;18] electrocardiogram recordings were performed to determine the effect of TC on heart rate and PR interval (Fig 1A and 1B). As has been observed clinically with fetuses in ICP, high levels of the bile acid TC prolonged the time for impulse propagation through the atria and AV node (PR interval) in fetal hearts. It was also observed that treatment with TC 400 μM caused a significant prolongation of PR interval in fetal hearts from baseline compared to vehicle treatment (Fig 1C and 1D). However, this slowing of conduction was prevented by UDCA co-treatment. Overall, no significant change was observed in heart rate following TC treatment. Importantly, no significant changes were observed in PR interval or heart rate when UDCA was applied alone (S1 Fig).

To investigate the mechanism behind the fetus-specific complications observed in ICP, the effects seen in the fetal hearts with TC and UDCA, were also investigated in the maternal hearts. In contrast to the effects observed in the fetal hearts, the high concentration of TC (400 μM) did not induce any significant changes in PR interval or heart rate in the maternal hearts (Fig 1E and 1F). Importantly, as with the fetal hearts, treatment with UDCA alone did not alter PR interval in the maternal hearts.

### UDCA protects against ventricular conduction slowing induced by TC

**Fig 2:**
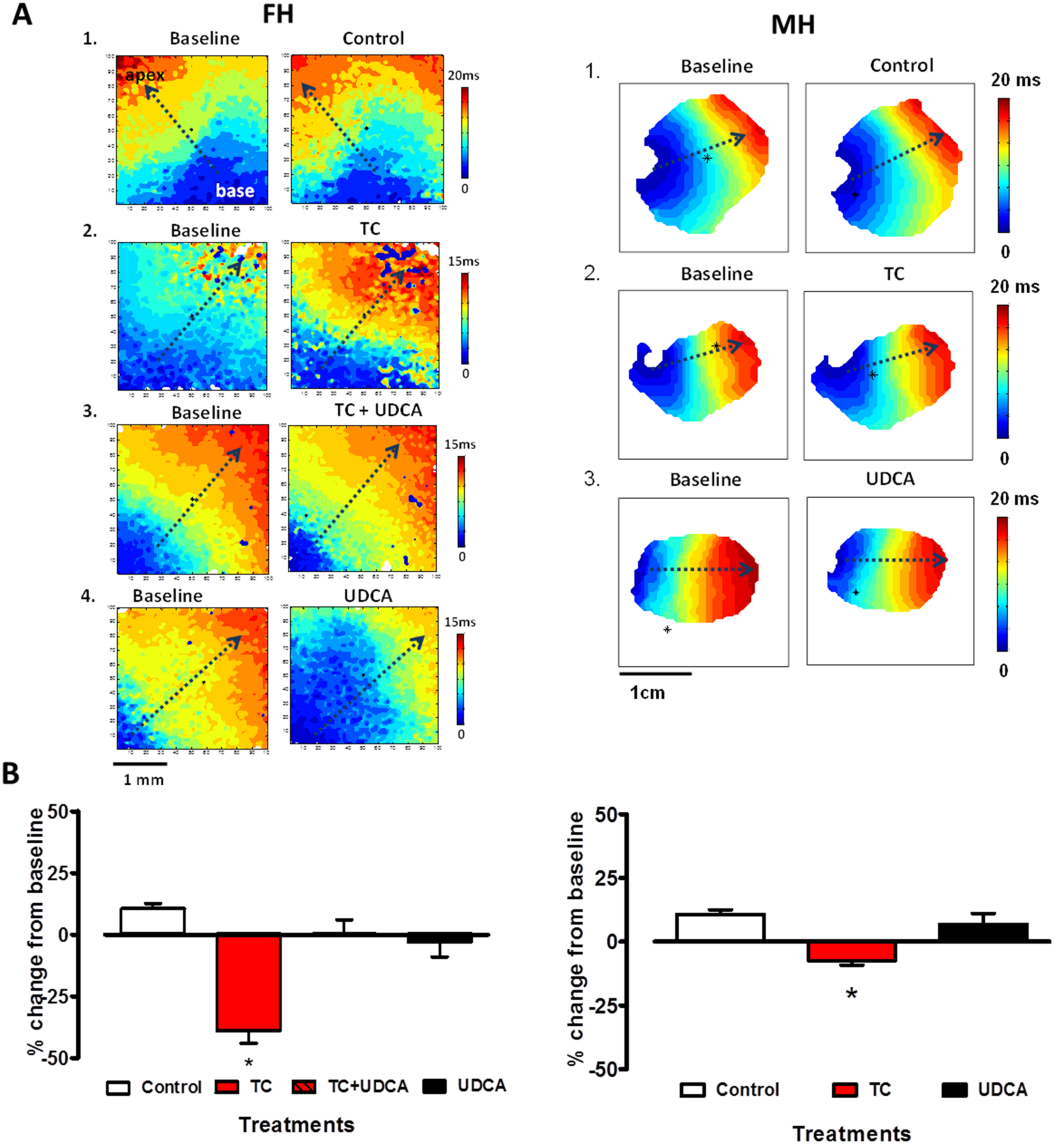
Co-treatment with UDCA protects against TC induced conduction velocity slowing in the fetal heart. A: Representative activation maps from each of the treatment groups in the fetal (FH) and maternal (MH) hearts (dashed arrows indicate direction of impulse propagation from point of stimulation). B: Plots of percentage change from baseline with control, TC 400 μM or TC 400 μM plus UDCA 1 μM treatments on conduction velocity in the fetal heart (*n* = 4–5 per group) and maternal heart (*n* = 3–4 per group). TC—taurocholic acid; UDCA—ursodeoxycholic acid. *P<0.05 One-way ANOVA and Bonferroni’s post hoc test to determine statistical significance.

The effect of TC on impulse propagation in the ventricles was investigated by optical mapping of the membrane potential (Fig 2A). In the fetal hearts, a statistically significant decrease in conduction velocity (CV) from baseline values was observed in hearts treated with TC 400 μM alone compared to hearts in the vehicle treatment group (Fig 2B). However, co-treatment of TC with 1 μM of UDCA was effective in protecting against such CV slowing. Reflecting the clinical safety of UDCA in fetal and maternal hearts,[19] treatment of hearts with UDCA alone did not produce any adverse changes in this study. Furthermore, a significantly smaller decrease in CV (−7.4±1.8%; *P*<0.05) was observed in the maternal hearts compared to the decrease in the fetal hearts (Fig 2A and 2B).

### UDCA protects against conduction slowing induced by calcium, but not sodium, channel blockers

**Fig 3:**
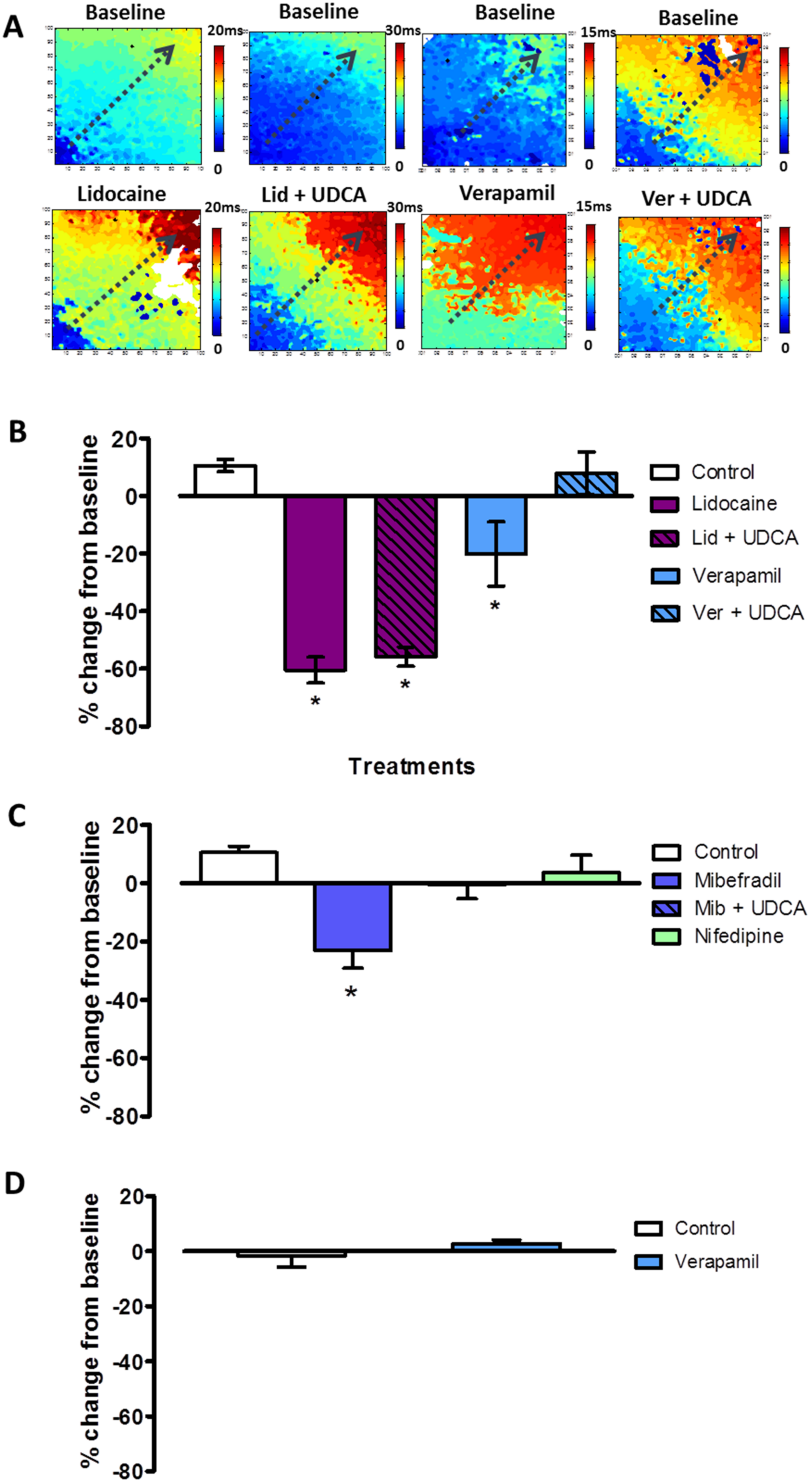
UDCA inhibited conduction velocity slowing induced by a calcium, but not a sodium, channel blocker. A: Representative activation maps from baseline and drug treatments in fetal hearts (dashed arrows indicate direction of impulse propagation from point of stimulation) B: Plots of percentage change from baseline following control; lidocaine 30 μM; lidocaine plus UDCA 1 μM; verapamil 1 μM and verapamil 1 μM plus UDCA 1 μM in the fetal hearts (*n* = 4–5 per group). C: Percentage change from baseline plots following control, mibefradil 1 μM, mibefradil 1 μM plus UDCA 1 μM and nifedipine 1 μM in the fetal model (*n* = 4–5 per group). D: Plots of percentage change from baseline following control and verapamil 1 μM treatment in the maternal hearts (*n* = 3 per group). *P<0.05 One-way ANOVA and Bonferroni’s post hoc test to determine statistical significance.

To understand the basis of the different responses in the fetal and maternal hearts, we evaluated the effect of a sodium channel blocker, lidocaine (30 μM), in fetal hearts. Lidocaine blocks Na^+^ channels and is known to cause conduction slowing in adult myocardium,[20] In the present study, lidocaine treatment also caused conduction slowing in fetal hearts (Fig 3A and 3B). This effect was not inhibited by UDCA co-treatment (−55.8±3.3%; *P*<0.05). The influence of the calcium component on CV slowing in both fetal and maternal hearts was then compared by treatment with a Ca^2+^ channel blocker, verapamil (1 μM). In the fetal heart, verapamil resulted in significant CV slowing, which was inhibited by co-treatment with 1 μM UDCA, unlike treatment with lidocaine (Fig 3B). When the same concentration of verapamil was applied to the maternal heart (Fig 3D), no change in CV was observed. These findings helped to suggest that the conduction slowing induced in the ventricles by TC is due to blockade of calcium ion channels and that UDCA protects against this slowing by modulation of calcium, and not sodium, channels.

### Different fetal and maternal responses are mediated by TC blockade of the T-type calcium channel subtype

Since Ca^2+^ channels are expressed in both adult and fetal hearts, we thus hypothesized that the effect of TC on the fetal heart is due to blockade of the T-type Ca^2+^ channel subtype current, which is specifically observed in developing hearts.[21] We therefore compared the effect on CV of two agents with higher selectivity for the cardiac T-type (mibefradil 1 μM) and L-type (nifedipine 1 μM) calcium channel subtypes in the fetal hearts. As shown in Fig 3C, treatment with mibefradil resulted in a significant CV decrease that was inhibited with the addition of UDCA; however, nifedipine treatment did not affect CV. Taken together, these results suggest that the slowing of ventricular impulse propagation in the fetal hearts by TC, and UDCA can be attributed to their modulation of the cardiac T-type calcium channel subtype, but as the expression of these channels is reduced in the ventricles of adult hearts, the same effect is not observed there.

### TC and UDCA effects on calcium current amplitude in neonatal and human featal cardiomyocytes

**Fig 4:**
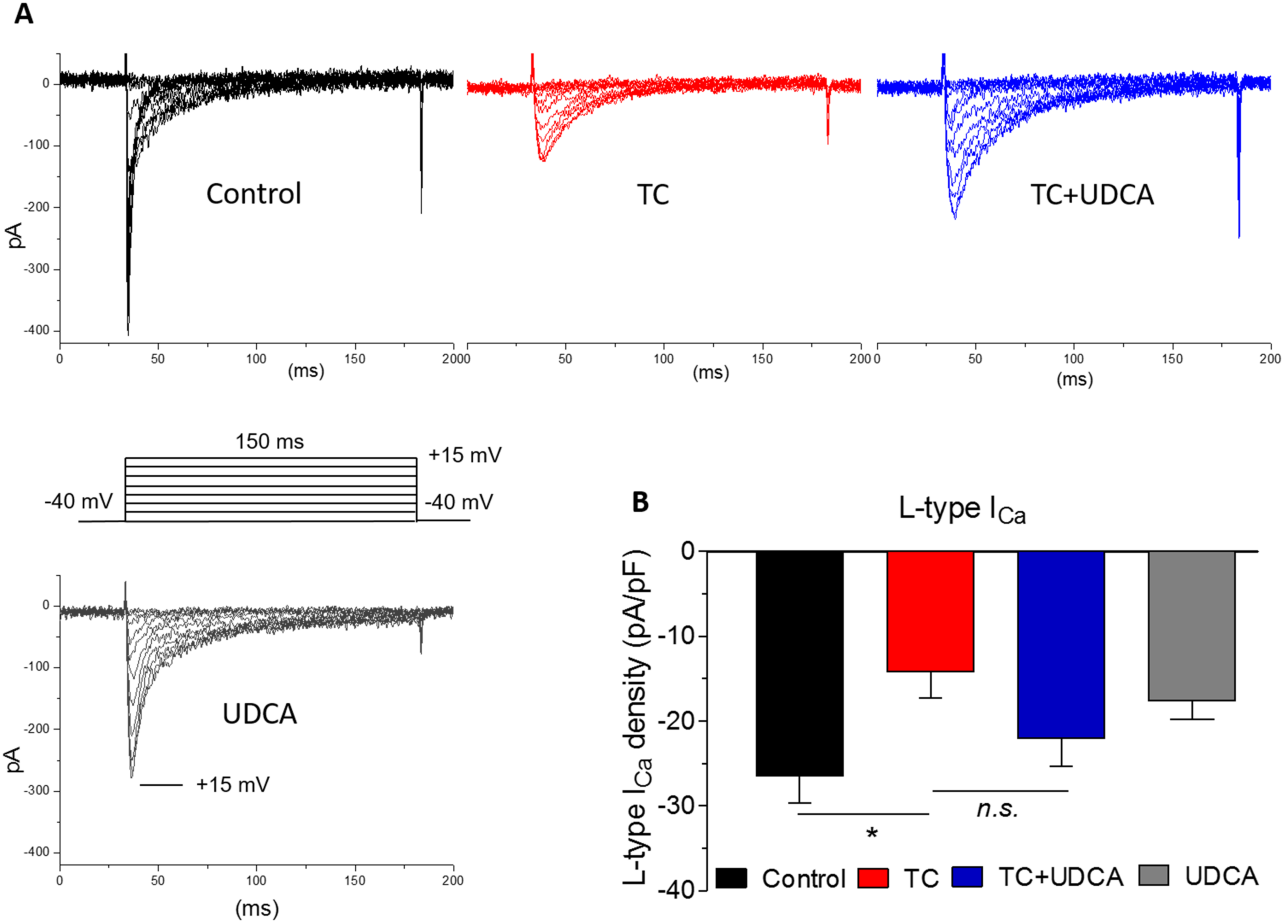
Effects of TC and UDCA on L-type whole-cell calcium currents from neonatal rat cardiomyocytes. A: Representative whole-cell current traces from neonatal rat myocytes for each condition. Inset: test pulse protocol during which currents were elicited from a holding potential of −40 mV to test potentials ranging from −40 to +65 mV over 150 ms in 5 mV increments. Current peaked between 10 and 20 mV. B: Mean peak L-type calcium current density (normalized to cell capacitance) from neonatal cardiomyocytes with vehicle (*n* = 13), in the acute presence of 100 μM TC (*n* = 7), 100 nM UDCA combined with TC (*n* = 12) and with UDCA alone (*n* = 8). *P<0.05 using One-way ANOVA and Bonferroni’s post hoc test.

TC had previously been shown to cause a decrease in *I*_Ca_ in adult rat cardiomyocytes and rabbit sinoatrial node preparations.[7;8] Therefore, we investigated the effects of TC and UDCA on calcium channel subtype currents in neonatal ventricular myocytes using the whole-cell patch-clamp technique (Fig 4A). TC 100 μM caused a decline in L-type calcium current amplitude, but as with the whole heart preparations, this effect was inhibited by co-administering 100 nM UDCA (Fig 4B).

**Fig 5:**
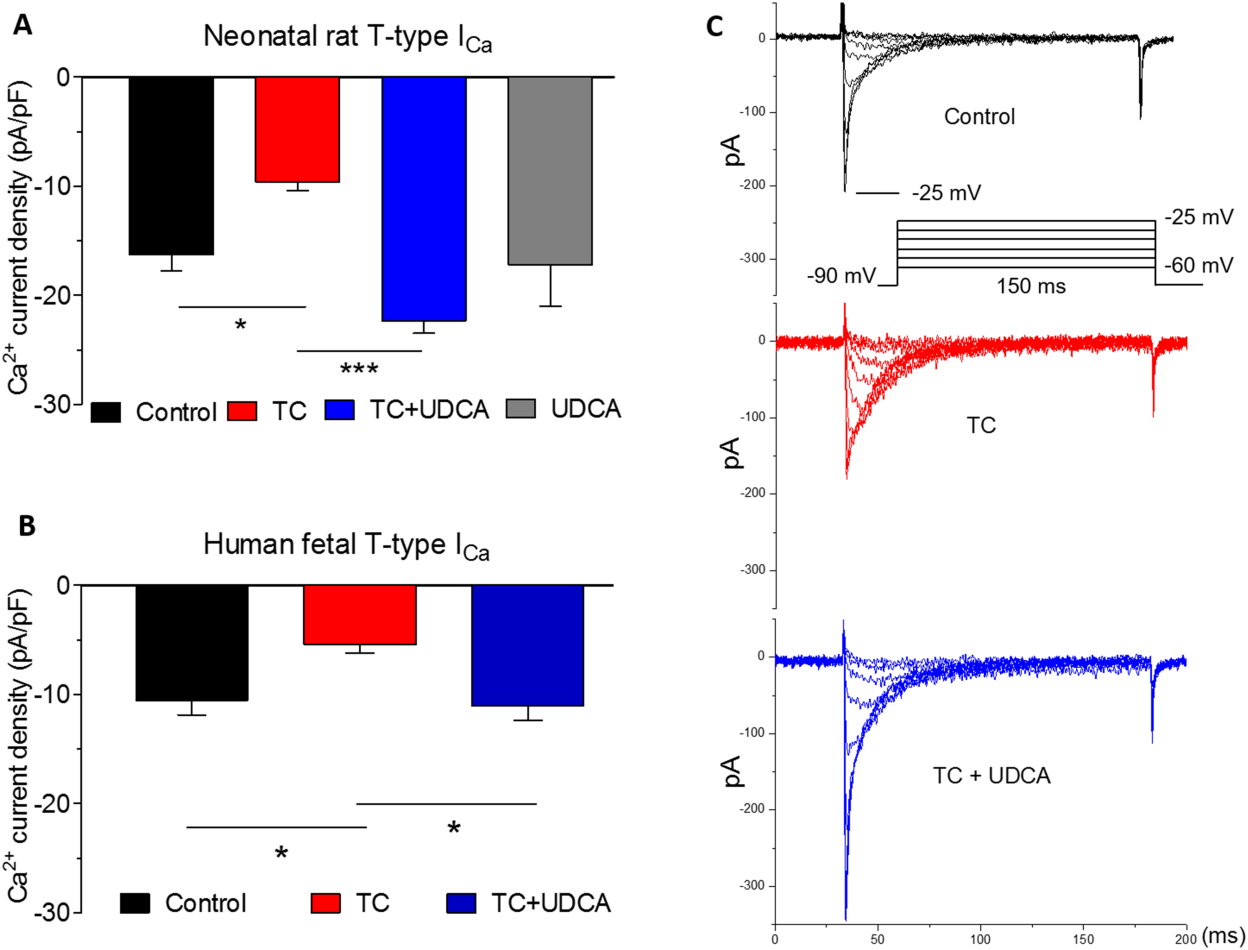
Effects of TC and UDCA on T-type calcium currents in neonatal rat and human fetal cardiomyocytes. A: T-type calcium current density in neonatal rat cardiomyocytes from control (*n* = 12), TC-treated (n = 14), 100 nM UDCA combined with TC (*n* = 10) and UDCA only treatments (*n* = 6). 200 nM nifedipine was included in all solutions to block the L-type current. Data are shown as mean with SEM. B: T-type calcium current density from human fetal cardiomyocytes with vehicle (*n* = 14), in the presence of TC (*n* = 12) and with 100 nM UDCA combined with TC (*n* = 11). C: Voltage-clamp protocol and representative whole-cell current traces from neonatal rat myocytes for each condition. Inset: Test pulse protocol during which currents were elicited from a holding potential of −90 mV to test potentials ranging from −60 to 50 mV over 150 ms, where peak current occurred at -25 mV *P<0.05, ***P<0.0001 using One-way ANOVA and Bonferroni’s post hoc test.

In terms of the *I*_Ca,T_, TC 100 μM caused a reduction in current amplitude compared to the vehicle-treated control group (−16±1.5 versus −9.6±1.8 pA/pF, P<0.05) in neonatal rat cardiomyocytes (Fig 5A). The same concentration of TC caused a significant reduction in *I*_Ca,T_ current density recorded in human fetal cardiomyocytes (−5.4±0.8 versus −10.5±1.4 pA/pF in control), which was reinstated to near control levels in the presence of UDCA. UDCA prevented the TC-induced reduction in the *I*_Ca,T_ density in both isolated human fetal (−11.0±1.4 versus −5.4±0.8 pA/pF, P<0.05) and neonatal rat ventricular myocytes (−22.3±1.1 versus −9.6±0.9 pA/pF, P<0.0001) (Fig 5A and 5B).

## Discussion

Abnormally high maternal serum bile acid concentrations occur in ICP, with fetal arrhythmia and possible intrauterine death being major consequences. UDCA has been tested in placebo-controlled clinical trials to evaluate whether its use is associated with a reduction in the adverse pregnancy outcomes (stillbirth and preterm labour) seen in ICP. UDCA has shown efficacy in improving circulating maternal bile acid concentrations, with a good safety and tolerability profile. Despite reported beneficial cardiovascular properties in several hepatic disorders, UDCA’s mode of action is currently unclear. With the use of an *in vitro* ICP model of the fetal and maternal heart, we demonstrate the anti-arrhythmic and cardio-protective capacities of UDCA. In *ex vivo* whole hearts, UDCA prevented detrimental slowing of electrical signal propagation, associated with high bile acid (TC) levels and as a result of pharmacological inhibition of T-type calcium channels. Importantly, these effects were fetal-heart-specific, without effects in the maternal heart model. In addition, we demonstrate at the single-cell level that UDCA is able to restore bile-acid-induced calcium current decline, by selectively targeting developmentally regulated T-type channels.

### UDCA is anti-arrhythmic in a whole-heart model of ICP

In the present study, administration of TC at a concentration similar to previously reported plasma concentrations in ICP,[1] resulted in significant slowing of impulses propagation through the AV node (PR interval prolongation) and ventricles in fetal hearts. These findings are in agreement with our previous studies which demonstrated pro-arrhythmic conduction slowing induced by TC in cellular model of the fetal heart.[10;15] It is also consistent with studies performed in human atrial trabeculae where TC is arrhythmogenic, prolonging the contractile refractory period in a concentration-dependent manner.[9]

In our models, similar to findings in previous reports, treatment with UDCA protected against the pro-arrhythmic effects of TC. By using intact hearts, we were able to build on the previous model by studying UDCA’s effects in the presence of other factors necessary for impulse propagation. Furthermore, the concentration of UDCA chosen for this study is comparable to plasma concentrations in humans following oral UDCA treatment.[22]

### UDCA protects against fetal arrhythmias by modulation of T-type calcium channels

Another notable finding in this study is the more pronounced effect of TC treatment on impulse propagation in the fetal hearts compared to the maternal hearts. At similar concentrations of TC, the significant decreases in CV observed in the atria and ventricles of fetal hearts were either minimal or absent in the maternal hearts. This finding is important, as it correlates with observations in ICP where cardiac complications are only observed in fetuses but not the mother[2] and would suggest fundamental differences in the mechanism of action of bile acids in the hearts of developing fetuses compared to those of fully developed individuals.

While *I*_Na_ is the main depolarizing current responsible for impulse propagation in healthy adult hearts, its role is significantly diminished in pacemaker tissues and development as well as in pathology where CV is slower.[23] In those cases, the proportion of the *I*_Ca_ impact can increase and its contribution to propagation can reach up to 50% of the total depolarizing current required for continuous impulse propagation.[24] To date, the role of *I*_Ca_ in developing hearts has not been well elucidated, however, with the aid of sodium and calcium channel blockers we were able to identify a greater influence of calcium ion channels on the speed of impulse propagation in developing hearts compared to mature hearts. This might explain why increases in plasma concentrations of bile acids such as TC, that are known to block calcium ion channels, result in more pronounced effects in the fetal than in the maternal heart.

Moreover, we demonstrated that the T-type Ca^2+^ channel plays a greater role in impulse conduction in fetal hearts compared to the L-type Ca^2+^ channel. This further explains the different fetal and maternal responses to elevation in bile acid concentrations. During development, both *I*_Ca,L_ and *I*_Ca,T_ are recorded in ventricular cardiomyocytes; however, there is a decline in the *I*_Ca,T_ with age.[21] Ferron and colleagues demonstrated that *I*_Ca,T_ is expressed in 18-day-old fetuses, and although reduced, it was still present in cardiomyocytes from one-day-old rats, like those used in this study, but absent in cells from 21-day old rats [25]. Characterization of the T-type calcium channel expression during development has been performed by other authors. Niwa and colleagues demonstrated that during mouse embryonic development the Ca_v_3.2 subtype underlies the functional I_Ca,T_ [26]. They demonstrated that its expression level decreased by approximately half between early (E9.5) and late (E18) embryonic stages and is almost absent in adult hearts. By the adult stage, Ca_v_3.1 level was found to be greater than that of Ca_v_3.2. Quantification of mRNA expression of Ca_v_3.1 and Ca_v_3.2 revealed that levels up to day 5 after birth were comparable to those at the prenatal stage but had decreased by 21 days after birth and in adults.[25] In the healthy adult ventricle, the *I*_Ca,T_ is almost absent in working myocardium of ventricles and atria and its expression is limited to cells of the conduction system, although expression levels can be elevated under pathological conditions, such as heart failure or hypertrophy.[27]

TC differentially acts on T- and L-type calcium currents in neonatal rat cardiomyocytes. *I*_Ca,T_ may have a greater impact on CV in fetuses when compared to *I*_Ca,L_, because *I*_Ca,T_ is low-voltage activated versus high-voltage activated *I*_Ca,L_. The activation range of *I*_Ca,T_ is positive to approximately -60 mV, whereas *I*_Ca,L_ activates positive to approximately -20 mV. Moreover, T-type Ca^2+^ channel steady-state availability is maximal for voltages less than -90 mV where they can work together with sodium channels and contribute to CV, whereas substantial close-state L-type channel inactivation is not observed for potentials as positive as approximately -40 mV. Thus, inhibition of *I*_Ca,L_ induced by TC should not affect CV as much as a similar (or greater) suppression of I_Ca,T_. A significant increase in *I*_Ca,T_, with TC and UDCA co-administration, was not seen in the case of *I*_Ca,L_, which indicates that enhanced Ca^2+^ entry through T-type channel subtype contributes to reinstatement of cardiac impulse speed. In addition, differential expression and function of T-type calcium channels in ventricular versus atrial myocytes may account for the effects seen at the whole-heart level. Ono and colleagues (2010) reported that during postnatal development, functional contribution of ventricular L-type channels exceeded that of T-type channels. On the other hand, atrial myocytes had comparable T- and L-type current densities at identical developmental stages[27]. It would therefore be of interest to evaluate the effects of TC and UDCA in atrial myocytes.

We also demonstrate that UDCA is able to prevent a significant reduction in CV slowing with calcium channel blockers, potentially by enhancing calcium entry through T-type channels. This lack of a complete *I*_Ca,T_ reversal by UDCA may suggest that other mechanisms are involved in CV slowing by these agents. We should also point out that the effects of TC are not likely to be limited to effects on T-type calcium channels. Previous work from our group and others has highlighted other targets for bile acids, like TC, in the heart, such as the muscarinic receptors and Na^+^/Ca^2+^-exchanger [9;11], which may additionally contribute to CV slowing. Further work will be required to demonstrate a clear link between the inhibition of T-type channels by TC and conduction slowing.

### Potential clinical applications of UDCA in cardiovascular conditions

UDCA has potential for use as an anti-arrhythmic agent for fetal arrhythmia and following myocardial infarction. A clinical trial in humans is underway to evaluate UDCA as an anti-arrhythmic agent post-myocardial infarction. If well-designed trials confirm its effectiveness in human *in vivo* studies, UDCA could be used within a small number of years.

A better understanding of the mechanism of action of UDCA could help to broaden its clinical application to the treatment of other cardiac conditions where potential benefits have been identified. In patients with chronic heart failure, for instance, treatment with UDCA has been shown to improve peripheral blood flow.[28] In heart transplant patients with cholestasis, UDCA treatment has helped to reduce the incidence of acute rejection.[29] In a double-blind, cross-over study in healthy volunteers, UDCA caused a reduction in diastolic blood pressure compared to controls.[30]

The findings of this study help to suggest potential applications for UDCA in the treatment of arrhythmias linked to changes in the expression of calcium channels, such as those seen in chronic cardiac infarction. It might also be beneficial in the treatment of long-term cardiomyopathies that are observed in cholestasis patients.[31] These findings also further highlight UDCA’s beneficial properties in the heart, as other studies have reported beneficial effects of UDCA treatment in infarction and ischaemia–reperfusion injury.[32;33] UDCA is also able to improve peripheral haemodynamics in patients.[34;35] UDCA also appears to be important for heart function in healthy individuals, as a study has shown that UDCA plasma levels are lower in atrial fibrillation patients than in healthy controls, suggesting that the balance between the plasma concentrations of UDCA and other bile acids can help to protect against arrhythmias.[9]

## Conclusions

In summary, we have shown that co-treatment of the bile acid TC with UDCA protects against cardiac impulse propagation slowing in the atria and ventricles of fetal hearts. We revealed that in fetal hearts, ventricular conduction depends on both sodium and calcium components. Following on from this, we demonstrated that UDCA protects against fetal arrhythmias by inhibiting the effect of TC on the *I*_Ca,T_. Thus, the fetus-specific effect of UDCA could be explained by targeting of *I*_Ca,T_, which is exclusively expressed in fetal hearts.

## Supporting information

S1 Fig Effect of UDCA on PR interval and heart in fetal and maternal hearts. A and B: Representative ECG overlay traces with timescale bars from fetal (*left column*) and maternal (*right column*) models. C and D: Plots of mean percentage change from baseline values in PR interval in control and UDCA 1 μM treated groups. E and F: Plots of mean percentage change from baseline values in heart rate in control and UDCA 1 μM treated groups.
(DOCX)
