# Supplementary material for: Ursodeoxycholic acid prevents ventricular conduction slowing and arrhythmia by restoring T-type calcium current in fetuses during cholestasis
Source: PLoS One. 2017 Sep 21;12(9):e0183167. doi: 10.1371/journal.pone.0183167 (PMC5608194; doi:10.1371/journal.pone.0183167)
Supplement: S1 Fig — A and B: Representative ECG overlay traces with timescale bars from fetal (left column) and maternal (right column) models. C and D: Plots of mean percentage change from baseline values in PR interval in control and UDCA 1 μM treated groups. E and F: Plots of mean percentage change from baseline values in heart rate in control and UDCA 1 μM treated groups. (DOCX) [file pone.0183167.s001.docx]

**Supplementary Materials and Methods**

**Animals**

All animals were cared for and housed in accordance with the United Kingdom Home Office Guide on Animals (Scientific Procedures) Act 1986 and local ethical guidelines. Animals were purchased from Harlan Laboratories, UK.

**Drugs**

All reagents used in this study were obtained from Sigma-Aldrich, UK. Sodium taurocholate, verapamil, mibefradil and lidocaine were dissolved in purified water while ursodeoxycholic acid (UDCA), nifedipine and Di-4-ANEPPS were dissolved in dimethyl sulfoxide (DMSO) at stock concentrations and then diluted to relevant concentrations for each study.

**Heart preparations**

To study the effects of bile acids one-day old neonatal rat (~7 grams, Sprague-Dawley) and adult female rat (200 - 400 g) hearts were used. Initial studies performed on neonatal rat hearts without aortic cannulation showed evidence of ischemia (results not shown) therefore it was necessary to develop the cannulation technique. To our knowledge this is the first published report of its use in a neonatal cardiac electrophysiology study. Hearts were cannulated as previously described.[1] Briefly, rats were anesthetised with 5% isoflurane-95% O_2_ and then decapitated. The chest cavity was opened and the heart removed and placed in heparinized cold (+4°C) cardioplegic solution of the following composition (in mM): NaCl 110, KCl 16, MgCl_2_ 16, CaCl_2_ 1.2 and NaHCO_3_ 10. Lung, thymus, and fat tissue were dissected and removed. A short section of aorta was attached to a cannula and the heart was retrogradely perfused with oxygenated (95% O_2_-5% CO_2_), constant temperature (37±1°C), modified Tyrode solution of the following composition (in mM): NaCl 128.2, KCl 4.7, NaH_2_PO_4_ 1.19, MgCl_2_ 1.05, CaCl_2_ 1.3, NaHCO_3_ 20.0 and Glucose 11.1. Hearts were superfused with the same solution used for retrograde perfusion and maintained at a similar constant temperature.

**Experimental protocol**

Hearts were randomly allocated to four treatment groups (for specific sample sizes for each group see figure legends below). Hearts were allowed to stabilize for an initial 15 min period before being treated for a further 15 min with either vehicle or drugs.

As previous work has shown that human fetuses in ICP have a longer electrocardiogram (ECG) PR interval compared to those from healthy mothers,[2] this study evaluated the effect of bile acids on PR interval in the *ex vivo* models. Hearts were treated with increasing concentrations of TC (40, 100 and 400 µM) or vehicle. Following on from this the effect of TC 400 µM with UDCA 1 µM or UDCA 1 µM alone were also evaluated. Comparison studies in the maternal hearts with TC were also performed at similar concentrations. During the stabilisation and treatment periods, ECG was recorded in the neonatal hearts at a sampling frequency of 1 kHz using a two-lead placement across the heart. Analysis was performed using Clampfit 10 (Molecular Devices, USA). Similarly, ECG recording was performed in the maternal hearts throughout the treatment duration at a frequency of 1 kHz using a PowerLab data acquisition system (AD Instruments, Sydney, Australia) and analysed using Labchart 7 pro software (AD Instruments, Spechbach, Germany).

**Optical mapping studies**

Optical mapping was performed as previously described.[3] Briefly, hearts were stained by coronary perfusion with the voltage-sensitive dye Di-4-ANEPPS during the stabilisation period. Neonatal hearts were stained at concentrations of 20-30 µM while in the adult hearts adequate staining was achieved at approximately 5 µM.

Hearts were treated with vehicle, TC 400 µM, TC 400 µM with UDCA 1 µM or UDCA 1 µM alone. Following on from this, studies were performed using standard agents using a similar protocol at the following concentrations (in µM): verapamil 1, mibefradil 1, lidocaine 30, and nifedipine 1. To assess the effects of drugs on conduction velocity (CV), hearts were excited with light (530-40 nm) from a 150W xenon lamp (Cairn Research, UK). Emitted light from the preparation was passed through a long pass emission filter (>600 nm) (Thorlabs) before reaching the camera. A MiCAM Ultima-L CMOS camera (SciMedia, CA, USA) with high spatial (100 x 100 pixels) and temporal (1,000 frames/s) resolution was used for recording.

Following the stabilisation and treatment periods, pacing electrodes were placed at the base of the ventricle and hearts were paced at 5 Hz. Transmembrane voltage transients were recorded for 2 second periods each time. Activation pattern maps were reconstructed using a customized MATLAB R2013a program (The MathWorks, Inc., MA, USA).[4] Signals were filtered using a low-pass Butterworth filter algorithm (fc = 200 Hz). Maximum upstroke derivative (dV/dt_max_) was calculated for each action potential using the normalized optical signal and its derivatives. Activation maps were constructed from activation times, which were determined from dV/dt_max_.

**Whole-cell patch-clamp recordings in cardiomyocytes**

Cardiomyocytes were isolated from one day old neonatal rat and human fetal hearts using enzymatic digestion as previously described.[5] Human fetal hearts were obtained from surgical terminations of pregnancy at 12 – 17 weeks of gestation, which were terminated due to scan or genetic abnormalities, after prior consent of the mother using Biobank ethical approval. Cells were incubated in M199 medium supplemented with 5% fetal bovine serum for two days before studies. The effect of vehicle, TC (100 μM) or TC (100 μM) plus UDCA (100 nM) on calcium current (*I*_Ca_) characteristics were studied using the whole-cell patch-clamp technique. *I*_Ca_ was recorded using an Axoclamp 200-B amplifier and Clampex data acquisition software (Axon Instruments, USA). L-type *I*_Ca_ (*I*_Ca,L_) was recorded over 150 ms from a holding potential of -40 mV to a test potential ranging from -40 to +65 mV in 5 mV increments. Current amplitude at +15 mV was taken as peak current. T-type *I*_Ca_ (*I*_Ca,T_) was recorded in response to test potentials ranging between -60 to +50 mV from a holding potential of -90 mV. Nifedipine was included in the control solution to block any residual T-type current. Results were analysed using Origin and Clampfit software. Peak current amplitude for each cell was normalised to cell capacitance and termed calcium current density.

**Statistical analysis**

Data were analysed and plotted using GraphPad Prism 5 software. Results are presented as percentage change values (mean ± SEM). Statistical analysis was carried out comparing treatment to control using 1-way ANOVA and Dunnett’s post hoc test. P values of less than 0.05 were considered to be statistically significant.

**S1 Fig. Effect of UDCA on PR interval and heart in fetal and maternal hearts.** A and B: Representative ECG overlay traces with timescale bars from fetal (*left column*) and maternal (*right column*) models. C and D: Plots of mean percentage change from baseline values in PR interval in control and UDCA 1 µM treated groups. E and F: Plots of mean percentage change from baseline values in heart rate in control and UDCA 1 µM treated groups.

Reference List

1. Glukhov AV, Flagg TP, Fedorov VV, Efimov IR, Nichols CG (2010) Differential K(ATP) channel pharmacology in intact mouse heart. J Mol Cell Cardiol 48: 152-160. S0022-2828(09)00369-1 [pii];10.1016/j.yjmcc.2009.08.026 [doi].

2. Strehlow SL, Pathak B, Goodwin TM, Perez BM, Ebrahimi M, Lee RH (2010) The mechanical PR interval in fetuses of women with intrahepatic cholestasis of pregnancy. Am J Obstet Gynecol 203: 455. S0002-9378(10)00678-2 [pii];10.1016/j.ajog.2010.05.035 [doi].

3. Glukhov AV, Fedorov VV, Anderson ME, Mohler PJ, Efimov IR (2010) Functional anatomy of the murine sinus node: high-resolution optical mapping of ankyrin-B heterozygous mice. Am J Physiol Heart Circ Physiol 299: H482-H491. ajpheart.00756.2009 [pii];10.1152/ajpheart.00756.2009 [doi].

4. Lou Q, Glukhov AV, Hansen B, Hage L, Vargas-Pinto P, Billman GE *et al*., (2013) Tachy-brady arrhythmias: the critical role of adenosine-induced sinoatrial conduction block in post-tachycardia pauses. Heart Rhythm 10: 110-118. S1547-5271(12)01021-1 [pii];10.1016/j.hrthm.2012.09.012 [doi].

5. Schultz F, Hasan A, Alvarez-Laviada A, Miragoli M, Bhogal N, Wells S *et al.*, (2016) The protective effect of ursodeoxycholic acid in an in vitro model of the human fetal heart occurs via targeting cardiac fibroblasts. Prog Biophys Mol Biol 120: 149-163. S0079-6107(16)00005-5 [pii];10.1016/j.pbiomolbio.2016.01.003 [doi].
